# Correspondence of Neutralizing Humoral Immunity and CD4 T Cell Responses in Long Recovered Sudan Virus Survivors

**DOI:** 10.3390/v8050133

**Published:** 2016-05-11

**Authors:** Ariel Sobarzo, Spencer W. Stonier, Andrew S. Herbert, David E. Ochayon, Ana I. Kuehne, Yael Eskira, Shlomit Fedida-Metula, Neta Tali, Eli C. Lewis, Moses Egesa, Stephen Cose, Julius Julian Lutwama, Victoria Yavelsky, John M. Dye, Leslie Lobel

**Affiliations:** 1 Department of Microbiology, Immunology and Genetics, Faculty of Health Sciences, Ben-Gurion University of the Negev, Beer-Sheva 84105, Israel; tautau.ariel@gmail.com (A.S.); askiray@gmail.com (Y.E.); fedida.metula@gmail.com (S.F.-M.); netatali20@gmail.com (N.T.); 2 Virology Division, U.S. Army Medical Research Institute of Infectious Diseases, 1425 Porter St., Fort Detrick, Frederick, MD 21701, USA; spencer.w.stonier2.ctr@mail.mil (S.W.S.); anderw.s.herbert.ctr@mail.mil (A.S.H.); aikuehne@aol.com (A.I.K.); 3 Department of Clinical Biochemistry and Pharmacology, Faculty of Health Sciences, Ben-Gurion University of the Negev, Beer-Sheva 84105, Israel; davidochayon@gmail.com (D.E.O.); lewis@bgu.ac.il (E.C.L.); 4 Medical Research Council/Uganda Virus Research Institute, Uganda Research Unit on AIDS, Entebbe P.O. Box 49, Uganda; m.c.egesa@gmail.com (M.E.); stephen.cose@lshtm.ac.uk (S.C.); 5 Department of Medical Microbiology, School of Biomedical Sciences, Makerere University College of Health Sciences, Kampala P.O. Box 7072, Uganda; 6 London School of Hygiene & Tropical Medicine, Keppel Street, London WC1E 7HT, UK; 7 Department of Arbovirology, Emerging and Re-Emerging Infection Uganda Virus Research Institute, Entebbe P.O. Box 49, Uganda

**Keywords:** Ebola survivors, memory immunity, neutralizing antibodies, cellular immunity

## Abstract

Robust humoral and cellular immunity are critical for survival in humans during an ebolavirus infection. However, the interplay between these two arms of immunity is poorly understood. To address this, we examined residual immune responses in survivors of the Sudan virus (SUDV) outbreak in Gulu, Uganda (2000–2001). Cytokine and chemokine expression levels in SUDV stimulated whole blood cultures were assessed by multiplex ELISA and flow cytometry. Antibody and corresponding neutralization titers were also determined. Flow cytometry and multiplex ELISA results demonstrated significantly higher levels of cytokine and chemokine responses in survivors with serological neutralizing activity. This correspondence was not detected in survivors with serum reactivity to SUDV but without neutralization activity. This previously undefined relationship between memory CD4 T cell responses and serological neutralizing capacity in SUDV survivors is key for understanding long lasting immunity in survivors of filovirus infections.

## 1. Introduction

Ebolavirus is a member of the *Filoviridae* family and the cause of viral hemorrhagic fever disease [1]. Studies that examined the pathogenesis of Ebolavirus infection in humans indicate that recovery is largely dependent upon, and associated with, the development of both cell-mediated and humoral immune responses [2,3,4,5]. Ebolavirus infection triggers the release of cytokines and chemokines, including interleukin (IL)-1β, IL-6, IL-8, IL-10, interferon (IFN)-γ, monocyte chemoattractant protein (MCP)-1, and IFNγ-inducible protein (IP)-10 [6,7,8]. In addition, evidence from studies that examined survivors and asymptomatic cases demonstrated the presence of significant levels of virus-specific IgM and IgG associated with a temporary, early and strong inflammatory response [5,9,10].

Prior to the recent outbreak in West Africa [11,12], one of the largest known outbreaks of ebolavirus, SUDV, occurred in Gulu, Uganda in 2000–2001, resulting in 425 cases and 224 fatalities [13]. The causative agent of this outbreak was named the Sudan virus (SUDV). Studies of the survivors of this outbreak indicate that the composition of survivor memory immune responses includes pro-inflammatory cytokine responses and antibody responses to SUDV antigens [14,15]. Further work has also demonstrated that a persistent humoral memory immune response with neutralization capacity was not present in all survivors of this cohort group and that a complete lack of memory humoral immunity was also observed in many survivors [16]. However, previous experiments that characterized SUDV survivor immune responses did not specifically measure antiviral memory T cell responses and could not determine the provenance of the cytokines being measured [15]. To address this, we obtained fresh whole blood samples from survivors of the Gulu SUDV outbreak, along with uninfected control individuals, and performed whole blood stimulation with SUDV antigens. The induced cytokine responses of memory T cells were studied by flow cytometry, coupled with multiplex ELISA to measure secreted cytokines and chemokines in supernatants of stimulated samples. Additionally, SUDV-specific IgG levels and SUDV-specific neutralization capacity were also assessed in matched serum samples. The results demonstrated a previously undefined correspondence between memory CD4 T cell responses and serological neutralizing capacity in SUDV survivors. Furthermore, survivors with significant serological immunoreactivity to ebolavirus antigens, but lacking serological neutralization capacity, failed to demonstrate this correspondence. As a result, this study reveals a potential linkage between only the neutralizing arm of the humoral immune response and cellular immunity in ebolavirus survivors.

## 2. Materials and Methods

### 2.1. Study Design

Subjects included confirmed survivors, according to patients PCR and ELISA results, from the SUDV outbreak of 2000–2001 in Gulu district, Uganda [17], and healthy local community members that were not infected. Study participants were not related.

### 2.2. Ethics Statement

The study was approved by the Helsinki committees of the Uganda Virus Research Institute in Entebbe, Uganda (reference number GC/127/13/01/15), Soroka Hospital, Beer-sheva, Israel (protocol number 0263-13-SOR) and the Ugandan National Council for Science and Technology (UNCST) (registration number HS1332). Written informed consent as well as a personal health questionnaire was completed for each subject.

### 2.3. Sample Collection

Whole blood samples were obtained by routine antecubital venipuncture. Samples were directly aspirated into sterile vacutainers containing freeze-dried sodium heparin (final heparin concentration 14.3 units/mL, (Becton Dickinson, Franklin Lakes, NJ, USA). and kept at 4 °C until assayed. Assays were initiated approximately 6 h after being collected and 2 h after the samples were processed.

### 2.4. Antigens and Stimulations

Stimulation assay antigen included irradiated, sucrose gradient purified, SUDV (Gulu isolate) [16]. A lectin from *Phaseolus vulgaris* Leucoagglutinin, PHA-L, (Sigma-Aldrich, Rehovot, Israel) was used as a positive control for cell stimulation. For ELISA assays, irradiated SUDV (Gulu isolate), recombinant SUDV GP_1-649_, and total 293T cell lysate that expressed a given recombinant SUDV protein (NP, VP30, VP35 and VP40) were used as the capture antigens. Construction of the recombinant SUDV viral gene expression vectors and production of irradiated SUDV have been described previously [18].

### 2.5. Internal Control Sera

Internal human control sera for ELISA were previously described [16]. Positive controls for the detection of SUDV GP_1-649_ included murine monoclonal antibody 3C10 that targets SUDV GP_1-649_ [19].

### 2.6. Specific IgG Detection Assays

The levels of circulating anti-SUDV and anti-SUDV recombinant viral protein antibodies were determined by chemiluminescence ELISA, as previously described [15,16].

### 2.7. Normalization of Raw Data and Selection of Cut-off Values

Calculation of signal to noise (S/N) values for anti-SUDV recombinant proteins NP, VP30, VP35, and VP40 specific IgG was performed as previously described [18]. Calculation of S/N values was performed using the formula: (average result of control or test serum against cell lysate expressing the recombinant viral protein/average result of control or test serum against cell lysate not expressing the recombinant viral protein (mock antigen)). The cut-off value for IgG positive immune-reactivity was determined with a control set of negative sera and ten-fold stratified cross-validation analysis [20]. For the purified SUDV, and SUDV GP_1-649_ protein, raw ELISA data were converted to percent positivity (PP) of a high internal control antibody since we did not assay a mock antigen. Calculation of PP values, as well as the cut-off value, was performed as previously described [21]. Normalization of cytokine and chemokine expression levels in whole blood stimulation assays was performed by removing the background (unstimulated expression) for each respective stimulated sample. Determination of the cut-off value for positive cytokine stimulation was performed as follows: for each cytokine or chemokine, the average background value (no stimulation) was determined using all tested samples. Next, raw data for each stimulated sample was divided by the background value. Cut-off selection was then set using the average + 2XSD of the uninfected control group. Low positive (+), medium positive (++) and strong positive (+++) was determined as X < 2, 2 < X < 4, and 4 < X above the cut-off value, respectively.

### 2.8. Plaque Reduction Neutralization Test

Plaque reduction neutralization assays (PRNT_80_) were performed as previously described [22]. Neutralization titers were determined to be the last dilution of serum that reduced the amount of plaque by 80% compared with control wells. Plaque reduction neutralization assays were performed in the BSL-4 lab of United States Army Medical Research Institute of Infectious Diseases (USAMRIID) (Fort Detrick, Frederick, MD, USA).

### 2.9. Whole Blood Stimulation from SUDV Survivors and Healthy Volunteers

Whole blood stimulation was performed as previously described [15,23] with minor modifications. Freshly obtained, heparinized venous blood from SUDV survivors and healthy volunteers was aliquoted into 12 × 75 mm snap-cap polypropylene tubes under sterile conditions. Each blood sample from both survivor and control subjects was diluted 1:4 in RPMI-1640 (Roswell Park Memorial Institute medium) supplemented with 5% FCS (Fetal Calf Serum). SUDV antigen (10 µg/mL) was added to individual aliquots (1.0 mL final vol) and the cultures were incubated at 37 °C in a 5% CO_2_ humidified environment for 22 h. For the final four hours of incubation, whole blood cultures were supplemented with brefeldin A (eBioscience, San Diego, CA, USA) to trap intracellular cytokines. Following incubation, cells were pelleted and processed for flow cytometry analysis while culture supernatants were aspirated, transferred to new 1.5 mL tubes and frozen at −70 °C until further processing.

### 2.10. Cytokine and Chemokines Detection Using Q-Plex™ ELISA-Based Chemiluminescent Assay

Levels of human cytokines IL-1β, IL-2, IL-4, IL-5, IL-6, IL-10, IL-13, IL-17, IL-23 IFNγ and TNFα and chemokines GROα, Eotaxin, I-309, IP-10, MCP-1 and MCP-2 were detected using Q-Plex technology (Quansys Biosciences, Logan, UT, USA) according to the manufacturer’s instructions. Readouts were obtained with a Quansys Imager (Quansys Biosciences) and results analyzed using the Q-View Software program (Quansys Biosciences). A human sL-Selectin Instant ELISA (eBioscience) was also performed according to the manufacturer’s recommended protocol, with the exception that culture supernatants were diluted 1:10, of which 50 μL was added to provide plates in duplicate.

### 2.11. Flow Cytometry Analysis

Following lysis of red blood cells, cells were washed in PBS and stained with Aqua live/dead dye (Life Technologies, Carlsbad, CA, USA). Prior to surface staining, cells were incubated with 1% mouse serum in flow staining buffer (eBioscience) to block nonspecific binding. Surface markers were stained for CD4, CD8, CD45RO, CD62L and CD3. Following fixation in 4% paraformaldehyde (BioLegend, San Diego, CA, USA), cells were permeabilized in 1× perm/wash (eBioscience) and incubated with antibodies against IFNγ and TNFα. Cells were stored in flow staining buffer at 4 °C prior to acquisition on an LSR II (BD Biosciences, San Jose, CA, USA). Flow cytometry data was analyzed in FlowJo (Tree Star, Ashland, OR, USA) and Excel (Microsoft, Redmond, WA, USA) and graphed in GraphPad Prism (GraphPad Software Inc., LA Jolla, CA, USA).

### 2.12. Statistical Analysis

Statistical analyses were performed using GraphPad Prism software 6.01 (GraphPad Software, Inc.). Correlation analysis was assessed by the Spearman nonparametric test. Differences in cytokine values between study groups were assessed by analysis of variants (ANOVA) and Wilcoxon rank sum test; *p*-values represent 2-sided *p*-values, and *p*-values < 0.05 were considered statistically significant.

## 3. Results

### 3.1. Cohorts and Blood Samples

Whole blood samples were obtained from 15 survivors of the SUDV outbreak in Gulu and five from uninfected members of the Gulu community, which served as controls. Samples were collected approximately 12 years post infection and all within 3 h of each other. All subjects were healthy and reported a lack of autoimmune diseases, cancer and past hospitalizations, unrelated to ebolavirus disease (EVD), suggesting a lack of confounding infections. Survivors reported uniform treatment (supportive care only) and symptoms during and after the acute illness.

### 3.2. Humoral Immune Responses to SUDV Proteins and Neutralization Profiles

**Figure 1:**
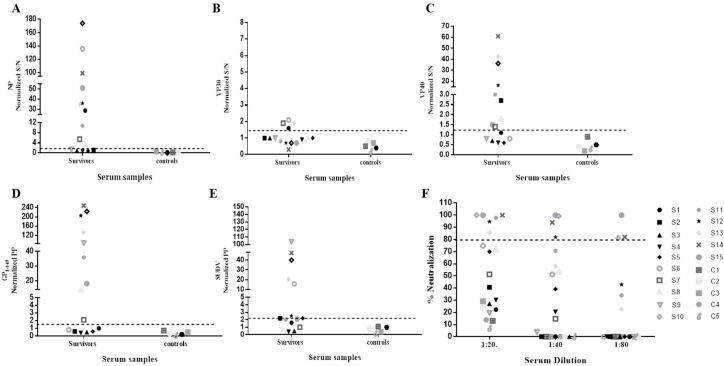
Summary of serological immune-reactivity and plaque reduction neutralization tests (PRNT_80_) of SUDV survivors and non-infected controls. (**A**–**E**) Serum samples from 15 SUDV survivors and 6 non-infected controls were tested by ELISA against individual recombinant SUDV viral proteins NP (**A**), VP30 (**B**), VP40 (**C**) and GP_1-649_ (**D**) as well as irradiated SUDV whole virus (**E**). Individual cut-off values for each tested viral protein or irradiated whole antigen is presented (dashed line). (**F**) Dilutions of serum samples (20, 40 and 80 fold) collected from SUDV survivors and uninfected controls were assayed for their ability to neutralize live SUDV in vitro under BSL4 conditions. The cut off value for the PRNT_80_ assay is denoted by the dashed line. S/N = Signal to Noise. PP = Positive percentage. S—SUDV survivor. C—Non-infected control. Percentage of neutralization is expressed as: 100 − [100 × (number of SUDV plaques obtained at given serum dilution/number of control SUDV plaques)].

We assessed the profile of IgG immune-reactivity using a custom made ELISA previously described [18]. Survivor serum samples were analyzed against SUDV recombinant proteins NP, VP30, VP40, GP_1-649_, and irradiated purified whole virus antigen. The results presented in Figure 1A–E) and Table 1 demonstrate that in the 15 SUDV survivors tested, 11 displayed positive antibody-reactivity to NP, nine to GP_1-649,_ and VP40, and four to VP30. Six survivors exhibited a positive antibody response against the irradiated SUDV whole purified virus antigen. Serum from uninfected controls displayed no reactivity to any of the viral antigens tested. A plaque reduction neutralization test (PRNT) was performed to determine the neutralization capacity of survivor sera at various dilutions (Figure 1F and Table 1). We defined neutralization as the ability of a given serum sample, regardless of dilution, to neutralize 80% of SUDV plaque formation (PRNT_80_) relative to controls. The results demonstrate that of the 15 SUDV survivors tested, sera from 6 survivors displayed a PRNT_80_ capacity. The remaining nine survivors and five uninfected controls were non-neutralizing by the applied definition.

### 3.3. Flow Cytometry

Flow cytometry analysis of IL-4, TNFα and IFNγ cytokine levels was performed following cell stimulation with SUDV inactivated whole antigen. Based on the humoral reactivity of serum samples observed in both the ELISA and PRNT_80_ assays (Table 1), we chose to group the survivors into the following categories for analytic purposes: those who have no immune-reactivity against SUDV GP_1-649_ or inactivated SUDV and were PRNT_80_ negative (Ab−/Neut−); those who react against SUDV GP_1-649_ and/or inactivated SUDV but were PRNT_80_ negative (Ab+/Neut-); and those who react against SUDV antigens and also have a PRNT_80_ capacity against live SUDV *in vitro* (Ab+/Neut+) (Table 1). Uninfected controls were also included and grouped separately.

**Figure 2:**
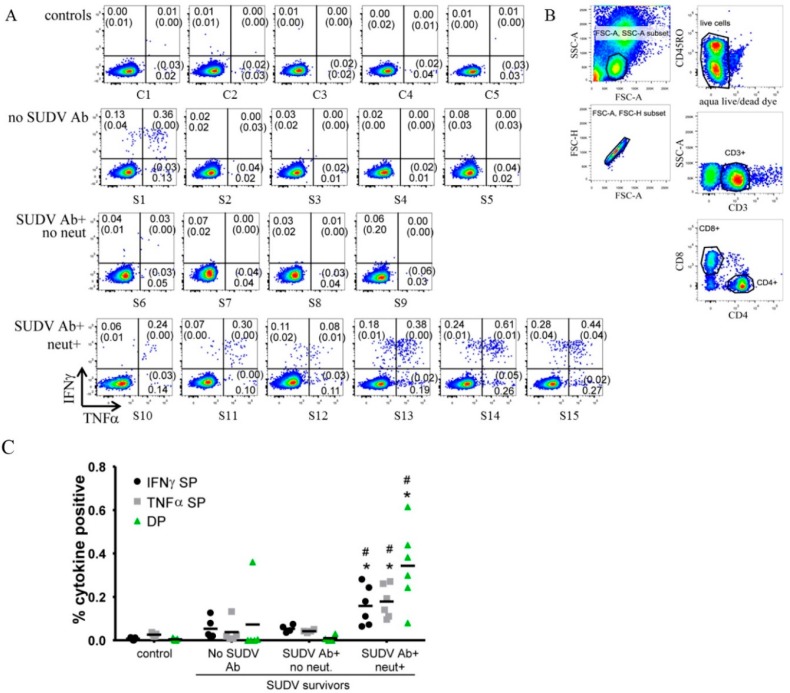
Flow cytometry results, gating strategy and analysis of whole blood from survivors and non-infected controls following SUDV whole antigen stimulation. (**A**) Plots depict IFNγ and TNFα cytokine responses in CD4 T cells following 22 h of stimulation with inactivated SUDV antigen, in non-infected controls, SUDV survivors without antibodies response, SUDV survivors with antibodies response and neutralization capability. Values in parentheses indicate the respective values for resting cultures that did not receive antigen; (**B**) Gating strategy for plots shown in (**A**); (**C**) Grouped column scatter plot showing the frequency of IFNγ single positive (SP), TNFα SP, or double positive (DP) events among CD4 T cells. Survivors are grouped according to the presence of IgG antibodies to SUDV antigen or GP_1-649_ and ability to neutralize live SUDV (Table 1). S: SUDV survivor; C: non-infected control; SP: single positive; DP: double positive; *: *p* < 0.05 SUDV Neut+ *vs.* SUDV Ab+ no Neut; #: *p* < 0.05 SUDV neut+ *vs.* no SUDV Ab.

Flow cytometry data showing IFNγ and TNFα expression by cells after stimulation with SUDV antigen for all survivors and controls is presented in Figure 2A. All plots shown are gated hierarchically as follows: lymphocytes (FSC-A *vs.* SSC-A), singlets (FSC-A *vs.* FSC-H), live cells (Aqua live/dead negative), CD3+ (CD3 *vs.* SSC-A), and CD4+ CD8− (Figure 2B). Resting values for each survivor or control are included in each quadrant in parentheses (Figure 2A). Cells from uninfected control samples did not express any IFNγ or TNFα in response to SUDV stimulation. One survivor in the SUDV Ab−/Neut− group had a diverse CD4 T cell response, comprised of IFNγ and TNFα-single positive as well as double positive cytokine-producing cells (Figure 2A). All other Ab−/Neut− and Ab+/Neut− survivors demonstrated an absence of cytokine production in response to SUDV stimulation. In stark contrast, survivors with PRNT_80_ serum capacity against SUDV *in vitro* all had multivariate cytokine responses (Figure 2A). The extent of response varied from survivor to survivor but overall, IFNγ and TNFα double-positive (DP) responses predominated. IFNγ single positive (SP), TNFα SP, and DP frequencies in the Ab+/Neut+ were significantly higher than all other groups for each subset of cytokine-producing cells (*p* < 0.05) (Figure 2C). Flow cytometry data of IL-4 cytokine levels following cell stimulation with SUDV inactivated whole antigen showed no detectable signals in both survivors and controls.

A correlation analysis between cytokine expression and neutralization activity (at 1:80 dilution) demonstrated a significant correlation between the cytokine and neutralization responses (Table 2). The correlation between neutralization and IFNγ, TNFα DP and TNFα SP cytokine responses was slightly higher than for IFNγ SP responses (Table 2). Only two of the 15 survivors had apparent CD8 T cell responses to SUDV (Supplemental Material Figure S1) consisting of IFNγ and TNFα expression. Due to the rarity of these responses, we were unable to do further analysis.

**Figure 3:**
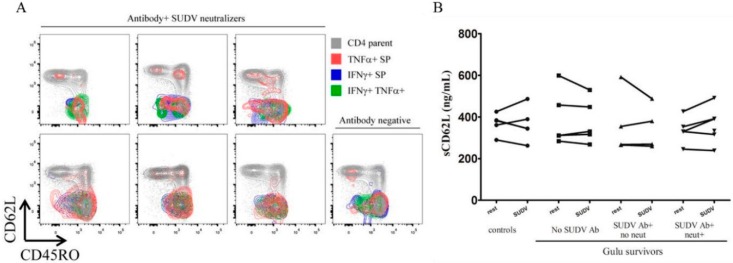
Memory subset analysis of CD4 T cell cytokine responses from survivors and non-infected controls following SUDV whole antigen stimulation. Subsets of CD4 T cell cytokine responses identified in Figure 3 were analyzed. (**A**) CK62L and CD45RO were used to identify T_CM_ (CN45RO+ CD62L+), T_EM_ (CD45RO+ CD62L−), naïve (CD45RO− CD62L+), and T_EMRA_ (CD45RO− CD62L−) T cell subsets. Each cytokine-producing cell population was overlaid upon the parent CD4 T cell population and color-coded to identify the expression pattern of CD45RO and CD62L; (**B**) sCD62l was measured in supernatants of whole blood resting cultures as well as with SUDV whole antigen. SP: single positive.

We also performed a memory subset analysis for survivors that had SUDV-specific CD4 T cell cytokine responses, regardless of whether or not they had SUDV-specific antibodies or serum neutralization capacity. We used CD62L and CD45RO in this analysis to identify central memory T cells (T_CM_, CD45RO+ CD62L+)_,_ effector memory T cells (T_EM_, CD45RO+ CD62L−), terminally-differentiated effector memory T cells (T_EMRA_ (CD45RO− CD62L−), and naïve T cells (CD45RO−, CD62L+). Each cytokine-producing subset was overlaid upon the parent CD4 T cell population for reference. Virtually all IFNγ SP and IFNγ, TNFα DP cells were in the T_EM_ subset (Figure 3A) with two survivors (upper right, lower left) that also had T_EMRA_ cells. Only TNFα SP cells were found in the T_CM_ subset (Figure 3A). As CD62L can be cleaved from the cell surface upon T cell activation, we performed an ELISA for soluble CD62L (sCD62L) in the supernatants of these cultures. We were unable to establish a pattern of increased sCD62L in cultures that corresponded to cytokine production, which would have indicated that CD62L had been cleaved due to T cell activation (Figure 3B). sCD62L levels were variable regardless of stimulation or resting culture for all controls and survivors (Figure 3B).

### 3.4. Cytokine and Chemokine Levels in Whole Blood Stimulation by Multiplex ELISA

In addition to flow cytometric analysis, multiplex ELISA was performed on supernatants to evaluate secreted cytokines and chemokines following whole blood stimulation using SUDV. During this assay, PHA-L (Phytohaemagglutinin Leukocytes) stimulation was used as a positive control for immune cell responses, and unstimulated samples served as baseline controls. The unstimulated control results demonstrated that SUDV survivors exhibited equivalent baseline levels of cytokines production compared to healthy, uninfected control patients, and PHA-L stimulation resulted in equally robust cytokine expression in all patient groups.

**Figure 4:**
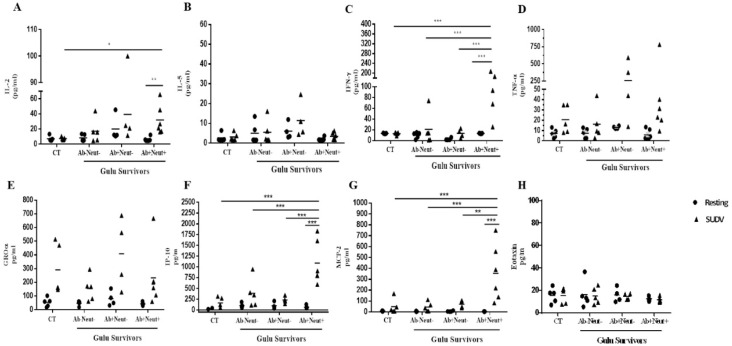
Multiplex DLISA of cytokines and chemokines secretion analysis of whole blood from survivors and non-infected controls following SUDV whole antigen stimulation. Cytokine and chemokine levels of IL-2 (**A**), IL-5 (**B**), IFNγ (**C**), TNFα (**D**), GROα (**E**), IP-10 (**F**), MCP-2 (**G**), and Eotaxin (**H**) were measured in the plasma supernatants of whole blood samples by multiplex ELISA following SUDV antigen whole blood stimulation and in the resting state (non-stimulated). Survivors are grouped according to the presence of IgG antibodies to SUDV antigen or GP_1-649_ and ability to neutralize live SUDV (Table 1). CT: non-infected controls, Ab: antibody, Neut: neutralizing; IL: interleukin, IFN: interferon, TNF: tumor necrosis factors, IP: IFNγ-inducible protein, MCP: monocyte chemotactic protein. Mean ± SEM, * *p* < 0.05, ** *p* < 0.01, *** *p* < 0.001.

Whole blood stimulation results with irradiated SUDV elicited significantly elevated secretion of IL-2, IFNγ, IP-10 and MCP-2 in Ab+/Neut+ survivors, relative to unstimulated, resting cultures (Figure 4A,C,F,G). IFNγ, IP-10 and MCP-2 secretion levels in Ab+/Neut+ survivors were also significantly increased relative to the other survivor groups tested. SUDV stimulation demonstrated no significant increase in secretion levels of IL-5, TNFα, GROα and Eotaxin. Multiplex ELISA results of IL-1β, IL-4, IL-6, IL-10, IL-17, IL-23, I-309, and MCP-1 showed no differences of cytokine levels or detectable cytokine signals in survivors and control groups. Further analysis between percent neutralization (at 1:80 dilution) and cytokine and chemokine secretion levels following stimulation revealed significant correlation to IL-2 (*p* = 0.0378), IFNγ (*p* = 0.0011), IP-10 (*p* = 0.0006), and MCP-2 (*p* = 0.0008) expression (Table 2). The complete multiplex ELISA results for the individual survivors and uninfected controls are presented in Table 3.

## 4. Discussion

Ebolavirus causes a severe hemorrhagic fever in humans resulting in a progressive and overwhelming disease [24]. Various studies support the notion that a robust, specific and adaptive immune response is required for survival from ebolavirus infection in humans [5,15,25]. These studies also suggest that protection from ebolavirus requires a balanced immune response with respect to both humoral and cell mediated immunity [3]. However the interplay between these immune components in long recovered ebolavirus survivors has not been fully characterized [4].

In this study, we demonstrated that 12-year post infection SUDV survivors present several distinctive profiles of immunity. Upon SUDV stimulation, significant differences in cytokine and chemokine expression levels and profiles were observed. A potentially important relationship between CD4 T cell cytokine expression and neutralizing humoral immunity was observed. Cytokines and chemokines, such as IL-2, IFNγ, TNFα, IP-10 and MCP-2, which are associated with CD4 T cell responses, and therefore indicative of T cell memory immunity, were significantly elevated in individual SUDV survivors with SUDV neutralizing serum activity. In contrast, no correlation was observed between CD4 T cell responses and the presence of non-neutralizing antibodies in survivors. Although this is yet to be proven, our thought is that one could make the hypothesis that the increased CD4 T cell population observed 12 years following the challenge could be indicative of a high viral load during infection driving a robust T helper response in these individuals. This could lead to enhanced class switch and affinity maturation to a more “potent” neutralizing phenotype of the antibody response. Alternatively, the long lasting CD4 T cell response and “potent” neutralizing response could also be a product of re-exposure to the same or “mimic” antigens that continue to mature the response. This could be due to the virus remaining in certain immune-privileged sites in the body as recently seen in the current West Africa outbreak [26], which may have been under appreciated in previous outbreaks.

The results described herein are in line with previous studies demonstrating that some long-recovered SUDV survivors can maintain persistent and strong IgG humoral immunity against SUDV, whereas others demonstrate a complete lack of memory immunity [14,16]. CD8 T cell responses were rare and may be due to a naturally small cell population or may be attributable to our use of whole antigens for stimulation. Alternatively, the lack of CD8 T cell responses may be observed due to the fact that the time point we analyzed is very distant from the survivor's infection. CD8 T cell responses were demonstrated recently in lymphocytes from EBOV survivors only months after infection using peptide stimulation [5]. Therefore, we plan to examine if CD8 T cell responses can be detected in these same SUDV survivors as well with SUDV specific peptide stimulation; however, this is beyond the scope of the current study.

Multiplex ELISA results following irradiated SUDV stimulation corresponded with flow cytometry data in that the survivor subgroup with serum neutralizing activity produced the greatest IFNγ response. In contrast to IFNγ secretion, the TNFα response demonstrated lower secretion levels. TNFα expression was more broadly detected among all survivors (Figure 4) and was not limited to the survivor group with serum neutralizing activity, as demonstrated by flow cytometry (Figure 2). It is unlikely that this difference is attributable to TNFα expression by CD8 T cells as the magnitude and breadth of CD8 T cell responses was low (supplemental Figure S1). Therefore, it is likely that the secreted TNFα detected by multiplex ELISA must come from a source other than T cells, although further evaluation is needed to validate this hypothesis. Further analysis of chemokine expression demonstrated a significant increase in levels of chemokines IP-10 and MCP-2 in SUDV survivors with serum neutralizing activity. Although the secretion of those chemokines can be attributed to various cell types [27], the elevated levels of IP-10 and MCP-2, which are known to play a role in chemotaxis and activation of T cells during viral infection [28], further support the presence of specific CD4 memory T cells in the subgroup of SUDV survivors with serum neutralizing activity.

## 5. Conclusions

Long-lived humoral immune responses have been characterized previously, but, to our knowledge, this is the first report detailing ebolavirus-specific CD4 T cell responses in long recovered human survivors. The results of our study indicate that long-lasting Sudan virus-specific CD4 T helper cells persist years after recovery in some survivors and, importantly, this phenotype may also correlate with serum neutralizing activity.

The data presented herein between long-lived cytokine, chemokine and humoral neutralization responses agrees with the notion that vaccines seeking to confer protection against filovirus exposure might benefit from eliciting both T cell and neutralizing antibody responses to provide complete and long-lived immunity. We do not know whether or not any of the survivors in this study may have had T cell responses that attenuated in the years since the outbreak. Nonetheless, our data in this specific cohort indicates that CD4 T cell responses and neutralizing antibody titers were linked in SUDV survivors in this window of time, even a decade post infection. What remains to be seen is if the relationship observed in this subset of patients, and in this particular cohort at this particular time point, is recapitulated in other survivor cohorts at varying time points following viral clearance. However, the study does make clear that clinical correlates and long term memory immune responses need to be further investigated in larger prospective human cohort studies of filovirus vaccine and therapeutic treatment modalities.

## Figures and Tables

**Table 1:** Summary of serological immune-reactivity and plaque reduction neutralization tests (PRNT_80_) results of SUDV survivors and non-infected controls.

	Serology					
	NP	VP40	VP30	GP_1–649_^1^	SUDV	PRNT_80_
C1	−	−	−	−	−	−
C2	−	−	−	−	−	−
C3	−	−	−	−	−	−
C4	−	−	−	−	−	−
C5	−	−	−	−	−	−
S1	++	−	+	−	−	−
S2	−	+	−	−	−	−
S3	−	−	−	−	−	−
S4	−	−	−	−	−	−
S5	−	−	−	−	−	−
S6	+++	−	+	−	+++	−
S7	+	−	+	+	−	−
S8	+++	+	−	+++	−	−
S9	++	+	−	+++	+++	−
S10	+++	+++	−	+++	+++	+++
S11	+++	++	−	+++	−	+
S12	+++	+++	−	+++	+	+
S13	+++	+++	+	+++	+++	+++
S14	+++	+	−	+++	+++	+++
S15	+++	+++	−	+++	−	++

^1^ A purified recombinant protein containing the 649 amino terminal amino acids of SUDV GP without the trans-membrane domain. S: Ebola survivors, C: non–infected controls. PRNT: plaque reduction neutralization test ELISA: (−) = ≤ cut-off value. (+) = < 2× cut off value, (++) = > 2× cut off value and < 4× cut off value, (+++) = > 4× cut off value. PRNT_80_: (+): neutralizes at 1:20 dilution, (++): neutralizes at 1:40 dilution, (+++): neutralizes at greater than 1:80 dilution.

**Table 2:** Correlation analysis between neutralization (1:80 dilution) and cytokine and chemokine secretion levels by multiplex ELISA and Flow cytometry following SUDV whole antigen stimulation in SUDV survivors and non-infected controls.

	Multiplex ELISA									Flow		
	IL-2	IL-5	IFNγ	TNFα	GROα	IP-10	MCP-2	Eotaxin		IFNγ	TNFα	TNFα + IFNγ
Spearman r	0.4672	0.0704	0.6892	0.2141	0.0778	0.6999	0.6860	0.2577		0.6714	0.7672	0.7561
*p* value (two side)	0.0378	0.7679	0.0011	0.3646	0.7442	0.0006	0.0008	0.2727		0.0012	<0.0001	0.0001

IL—Interleukin, IFN—Interferon, TNF—Tumor necrosis factors, IP—IFNγ-inducible protein, MCP—Monocyte chemotactic protein. *p* values < 0.05 were considered statistically significant.

**Table 3:** Summary of cytokines and chemokines secretion levels by multiplex ELISA following SUDV whole antigen stimulation in SUDV survivors and non-infected controls.

	Cytokine Secretion					Chemokine Secretion			
	IL-2	IL-5	IFNγ	TNFα		GROα	IP-10	MCP-2	Eotaxin
C1	−	−	−	−		−	−	++	−
C2	−	−	−	−		−	−	−	−
C3	−	−	−	−		−	−	−	−
C4	−	−	−	−		−	−	−	−
C5	−	−	−	−		−	−	−	−
S1	++	−	++	−		−	++	++	+
S2	−	−	−	−		−	−	−	−
S3	−	−	−	−		−	−	−	−
S4	+	−	−	−		−	−	+	−
S5	+	++	−	−		−	−	−	−
S6	−	−	−	−		−	−	−	−
S7	+	++	−	+++		+	−	−	−
S8	+++	+	+	+++		−	−	+	−
S9	+	−	−	−		−	−	+	−
S10	+	−	+	−		−	+	++	−
S11	+	−	+++	−		−	+	++	−
S12	+++	−	+++	−		−	+	+	−
S13	++	−	+++	−		−	++	+++	++
S14	++	−	++	+++		−	+++	+++	−
S15	+	−	ND	−		+	++	+++	−

S: Ebola survivors, C: non–infected controls, ND: not determined, IL: interleukin, IFN: interferon, TNF: tumor necrosis factors, IP: IFNγ inducible protein, MCP: monocyte chemotactic protein. (−) = ≤ cut-off value, (+) = <2× cut off value, (++) = >2× cut off value and < 4× cut off value, (+++) = >4× cut off value.

## Supplementary Files

Supplementary File 1
