# Supplementary material for: Correspondence of Neutralizing Humoral Immunity and CD4 T Cell Responses in Long Recovered Sudan Virus Survivors
Source: Viruses. 2016 May 11;8(5):133. doi: 10.3390/v8050133 (PMC4885088; doi:10.3390/v8050133)
Supplement: Supplementary File 1 [file viruses-08-00133-s001.pdf]

Supplemental Material: Correspondence of Neutralizing Humoral Immunity and CD4 T Cell Responses in Long Recovered Sudan Virus Survivors

Ariel Sobarzo, Spencer W. Stonier, Andrew S. Herbert, David E. Ochayon, Ana I. Kuehne, Yael Eskira, Shlomit Fedida-Metula, Neta Tali, Eli C. Lewis, Moses Egesa, Stephen Cose, Julius Julian Lutwama, Victoria Yavelsky, John M. Dye and Leslie Lobel

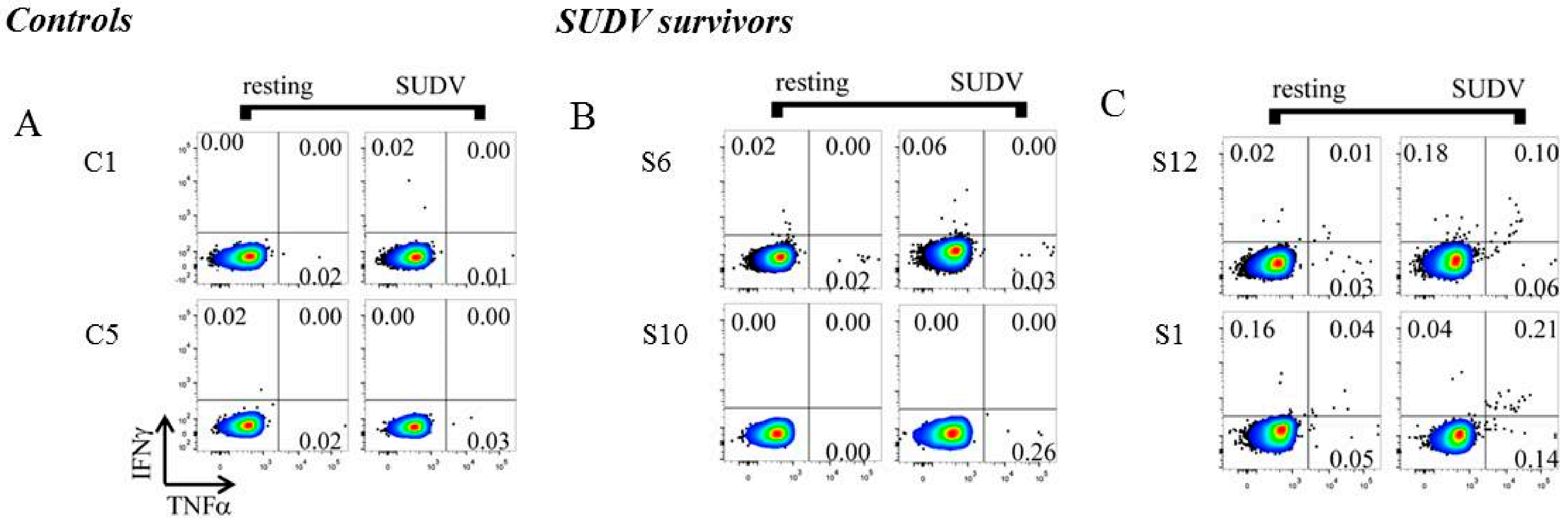

Figure S1. Flow cytometry CD8 T cell analysis of whole blood from survivors and non-infected controls following SUDV whole antigen stimulation. Represented plots depict IFNγ and TNFα cytokine responses in CD8 T cells following 22 h of stimulation with inactivated SUDV antigen of controls; C1 and C5 (**A**); and SUDV survivors with no detected CD8 T cell response; S6 and S10 (**B**) and detected S1 and S12 CD8 T cell response (**C**).

© 2016 by the authors; licensee MDPI, Basel, Switzerland. This article is an open access article distributed under the terms and conditions of the Creative Commons by Attribution (CC-BY) license (http://creativecommons.org/licenses/by/4.0/).
